# User activity recognition system to improve the performance of environmental control interfaces: a pilot study with patients

**DOI:** 10.1186/s12984-018-0477-5

**Published:** 2019-01-16

**Authors:** Arturo Bertomeu-Motos, Santiago Ezquerro, Juan A. Barios, Luis D. Lledó, Sergio Domingo, Marius Nann, Suzanne Martin, Surjo R. Soekadar, Nicolas Garcia-Aracil

**Affiliations:** 1 Miguel Hernández University of Elche, Av. Universidad w/n, Ed. Innova, Elche, 03202 Spain; 2 BJ Adaptaciones, St Mare de Déu del Coll, 70, Barcelona, 08023 Spain; 3 The Cedar Foundation, 1 Upper Lisburn Road, Belfast, BT10 0GW UK; 4 University Hospital of Tuebingen, Applied Neurotechnology Lab, Calwerstr. 14, Tübingen, D-72076 Germany; 5 Clinical Neurotechnology Laboratory, Neuroscience Research Center (NWFZ), Charité University Medicine Berlin, Charitéplatz 1, Berlin, 10117 Germany

**Keywords:** Environment control interface, Brain-computer interface, User intention prediction, Multimodal system, Brain injury, Spinal-cord injury

## Abstract

**Background:**

Assistive technologies aim to increase quality of life, reduce dependence on care giver and on the long term care system. Several studies have demonstrated the effectiveness in the use of assistive technology for environment control and communication systems. The progress of brain-computer interfaces (BCI) research together with exoskeleton enable a person with motor impairment to interact with new elements in the environment. This paper aims to evaluate the environment control interface (ECI) developed under the AIDE project conditions, a multimodal interface able to analyze and extract relevant information from the environments as well as from the identification of residual abilities, behaviors, and intentions of the user.

**Methods:**

This study evaluated the ECI in a simulated scenario using a two screen layout: one with the ECI and the other with a simulated home environment, developed for this purpose. The sensorimotor rhythms and the horizontal oculoversion, acquired through BCI2000, a multipurpose standard BCI platform, were used to online control the ECI after the user training and system calibration. Eight subjects with different neurological diseases and spinal cord injury participated in this study. The subjects performed simulated activities of daily living (ADLs), i.e. actions in the simulated environment as drink, switch on a lamp or raise the bed head, during ten minutes in two different modes, *AIDE* mode, using a prediction model, to recognize the user intention facilitating the scan, and *Manual* mode, without a prediction model.

**Results:**

The results show that the mean task time spent in the *AIDE* mode was less than in the *Manual*, i.e the users were able to perform more tasks in the *AIDE* mode during the same time. The results showed a statistically significant differences with *p*<0.001. Regarding the steps, i.e the number of abstraction levels crossed in the ECI to perform an ADL, the users performed one step in the 90% of the tasks using the *AIDE* mode and three steps, at least, were necessary in the *Manual* mode. The user’s intention prediction was performed through conditional random fields (CRF), with a global accuracy about 87%.

**Conclusions:**

The environment analysis and the identification of the user’s behaviors can be used to predict the user intention opening a new paradigm in the design of the ECIs. Although the developed ECI was tested only in a simulated home environment, it can be easily adapted to a real environment increasing the user independence at home.

## Background

It is estimated that one in six people in the world are diagnosed with a neurological disorder and this number is expected to rise considerable due to extensions of life expectancy [1]. A neurological condition is a damage to the brain, spinal column or nerves due to illness or injury such as spinal cord injury, acquired brain damage, stroke, motor neurons disease and locked in syndrome. Neurological disorders are considered the primary cause of disability in modern society [1, 2]. The debilitating consequences of neurological disorders include communication difficulties, impaired memory, inappropriate behavior, physical disability, restricted independence, social isolation and poor quality of life.

Assistive technologies aim to increase quality of life [3–6], reduce dependence on care giver [7] and reduce dependence on the long term care system [8]. Several studies have demonstrated the effectiveness in the use of environment control interfaces (ECI) for environment control or communication through voice commands [9], scan interfaces based on grid structure, eye tracking [10–12] or brain-computer interface (BCI) based on P300 [13], among others. These software platforms actively aid during the Activities of Daily Living (ADL) improving the independence both at home and outside. However, these platforms are based in a manual scan over the different abstraction levels of the ECIs and the scan speed only depends on the users familiarization with the system and the configuration of the grids over the different menus. Thus, introducing the user environment and behavior into this loop will help the navigation agility in the ECIs.

On the other hand, The progress of BCI research together with exoskeleton enables a person with motor impairment to interact with new elements of the environment [14, 15]. Thus, this progress will deliver new scenarios to BCI systems out of laboratories and move BCI into the domestic environment. The AIDE project^1^ aims to create new shared-control paradigm for assistive devices that integrates information from identification of residual abilities, behaviors, emotional state and intentions of the user on one hand and analysis of the environment and context factors on the other hand. In this context, a hybrid BCI model was chosen to control the ECI. It was developed as a fusion between non-invasive electroencephalography (EEG) and electrooculography (EOG) system [16]. The EEG records the sensorimotor rhythms (SMR) called event-related desynchronization (ERD) and event-related synchronization (ERS) during a motor imagery (MI) task [17] whilst the EOG records the horizontal oculoVersion (HOV).

This paper aims to evaluate the ECI developed under the AIDE project conditions, a multimodal system developed to assist people with acquired brain damage or neuro-degenerative diseases that need a wheelchair and has low or any upper limbs mobility in their ADLs, in a simulated environment able to detect the user intention through the environment analysis and the identification of the user’s behaviors, based on a conditional random fields (CRF) model [18]. Thus, the handling of the interface was studied in two different ways, with and without the prediction of the user’s intention. Users with neurological and muscular diseases and spinal cord injury (SCI) tested the system on a virtual home due to the early phase of the project.

## Methods

**Fig. 1:**
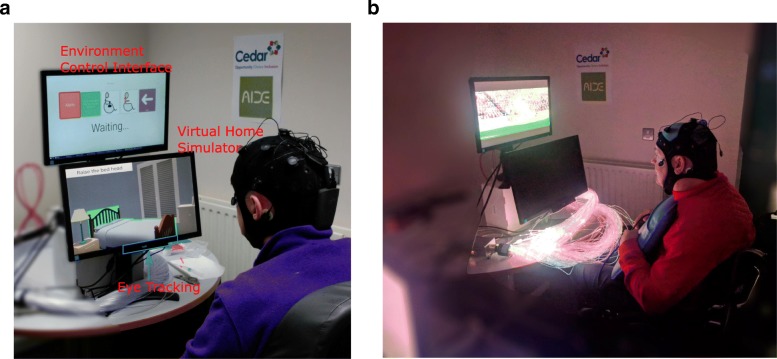
Experimentation setup. The experimental setup was composed by two screens layout: ECI & Virtual home simulator, and an eye tracking placed in the bottom screen. The users performed tasks in the simulated environment (**a**) and they also interacted with real elements (**b**)

This study evaluated the ECI in a simulated scenario under the AIDE project conditions, a multimodal interface able to analyze and extract relevant information from the environments as well as from the identification of residual abilities, behaviors, and intentions of the user. It consisted in a two screen layout: ECI and simulated room, with an EEG and EOG data acquisition system (see Fig. 1).

### Environment control interface

**Fig. 2:**
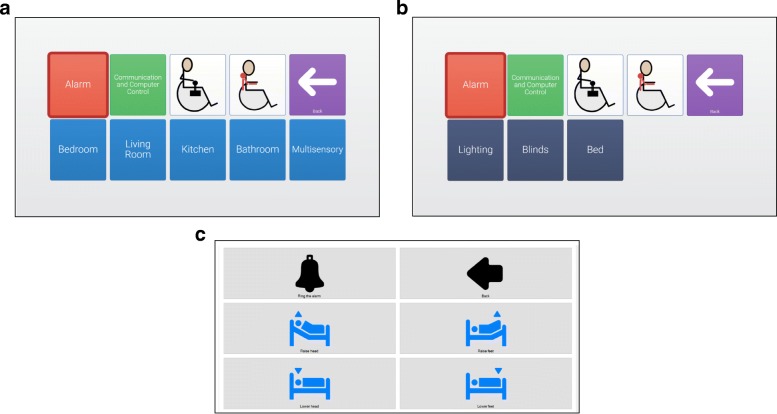
Environment Control Interface. The ECI had three different abstraction levels: 1) related with the room, 2) related to the activities that can be performed in a specific room and 3) related to the actions regarding a specific activity. An example menu of each level is shown in (**a**), (**b**) and (**c**) respectively

The environment control system used in this experimentation was based in two main software components: GRID3 from Smartbox^2^, a commercial augmentative and alternative communication (AAC) solution, and SHX, a specific developed software, presented in Fig 2. The ECI had three different abstraction levels: 1) related with the room (room menu), 2) related to the activities that can be performed in a specific room (activity menu), and 3) related to the actions regarding a specific activity (ADL menu). The jump between two consecutive abstraction levels will be named as *step*.

Levels one and two were specific grid sets, created in GRID3, to be used in the context of the experimentation. They include grids for the different rooms, communication, control a wheelchair and control an exoskeleton arm. In all grids, a color code has been used: red for the alarm; green for communication, computer control and digital leisure; white for wheelchair and arm control (not used in this experimentation); light blue for the rooms; dark blue for environmental control activity. The dark blue cells are referred to environmental control activities and linked Grid3 with the SHX application. The user could scan across the different cells, select one, and then confirm or cancel the selection using the chosen signals (EOG, EEG, eye tracking, etc.).

SHX is a custom build solution for environmental control management, level three. It allowed the user to easily configure and select the actions programmed for a specific activity. Different scenes for each of the possible activities in every room were created. Here too, the user could scan across the different cells, select one, and then confirm or cancel the selection using the chosen signals (EOG, EEG, eye tracking, etc.).

### Data acquisition

The acquisition of the brain activity was performed with eight solid-gel electrodes placed according to international 10-20 system placed at F3, C3, Cz, P3, T7 and Mastoid, a reference electrode was placed on C4 and the ground on FpZ. Furthermore, two electrodes were placed on the outer canthus of the eyes to the EOG signal recording. The EEG/EOG signal was acquired via Bluetooth through the Neuroelectrics amplifier (Enobio, Neuroelectrics, Barcelona, Spain). Skin/electrode resistance was kept below 12 kOhm.

A real-time SMR-based BCI was implemented using BCI2000, a freely distributed software for multipurpose standard BCI platform [19]. EEG and EOG were recorded at a sampling rate of 500 Hz, bandpass filtered at 0.4-70 Hz and pre-processed using a small Laplacian filter. Based on the maximum values for basal ERD, the ongoing EEG signal associated with the specified SMR rhythm frequency range (11-14 Hz) calculated from C3 electrode, a subject’s individual motor imagery discrimination threshold were set. The EOG discrimination thresholds were calculated regarding the average amplitudes of horizontal saccades. These individual parameters were obtained from the training session and used for later online BCI control [20].

### Prediction model

The proposed ECI combines the environmental information and context factors together with user’s behaviors in order to detect the user intention. Thus, the input information of the prediction model was a sequence of data, the user is moving and looking at the environment, that had to be labeled. In this context, different models were tested (time-delay neural networks, decision trees, hidden markov model (HMM)...) and the CRF model was chosen, showing the best results. The CRF model is a probabilistic model for segmentation and labeling sequence data. This discriminate model takes into account not only the current state but also the previous states to perform its prediction. A conditional model specifies the probabilities of possible label sequences given an observation sequence in contras of the generative models that make very strict independence assumptions on the observation, for instance conditional independence given the labels as HMM [21].

In our case, the inputs of the system were: localization, objects in the environment, object that the user is looking at, temperature of the room, brightness of the room and day time; the output was the ADL menu, i.e. the most probable action that the user wanted to perform that were directly linked with a specific ADL (see Table 1.

**Table 1:** Correlation between the ADLs and ADL menu name, in the third abstraction level

ADLs	ADL menu	ADLs	ADL menu
Open/close fridge	Drink or eat	Switch on/off air conditioner	Air conditioner
Open/close microwave	Drink or eat	Brushing teeth	Teeth
Eating task	Eat	Washing face	Face
Drinking task	Drink	Raise/lower the bed head	Bed
Switch on/off Music	Entertainment	Raise/lower the bed feet	Bed
Switch on/off PC	Entertainment	Open/close the blinds	Blinds
Switch on/off TV	TV	Switch on/off the light	Light

### Participants

Eight persons with different neurological pathology and spinal cord injury participated in this study (37±15 years old), their demographic and clinical characteristics are listed in (Table 2). The subjects were evaluated before the experiment with the barthel index [22]. All participants gave informed consent using their standard communication channel prior to participation in the study. The protocol was approved by the Office Research Ethics Northern Ireland - approval granted for project (15/NE/0384).

**Table 2:** Demographic and clinical characteristics of participants

ID Patient	Sex	Age	Diagnosis	Barthel score
1	Male	32	C4 SCI	4/20
2	Male	22	Duchenne Muscular Dystrophy	6/20
3	Male	55	Brain stem strokes	16.5/20
4	Male	30	C4/C5 SCI	2/20
5	Female	20	C6/C7 SCI	10/20
6	Male	58	Ischemic Stroke	19/20
7	Male	55	Multiple Systems Atrophy	5/20
8	Male	30	C6/C7 SCI	9/10

### Experimental protocol

Subjects were sitting in his/her own wheelchair in front of a table with two screens, as shown in Fig. 1. The screens were used to show the ECI and the virtual home simulator. Subjects used the AIDE multimodal interface, hybrid EEG EOG system, to online control the ECI and preform specific ADLs. Two modes were tested:

A) *MANUAL* mode: the user had to navigate through the three abstraction levels in order to accomplish the task showed in the virtual house. The objects related with the corresponding task were surrounded by a green color in the virtual house environment and the task appeared in the right top corner.
B) *AIDE* mode: in this mode the prediction model was used. The user had to look at the objects related to the specific ADL, showed like in the other mode, and, after the user’s intention prediction, the ECI directly jumped to the corresponding ADL menu. Then the user had to navigate like *MANUAL* mode. In case of wrong prediction, the user had to manually go back, the second abstraction level, and complete the corresponding ADL. The observed objects were online detected from the eye tracking Tobbi^3^ PCEye go, placed on the virtual environment screen, and the rest of the inputs were online simulated.

**Fig. 3:**
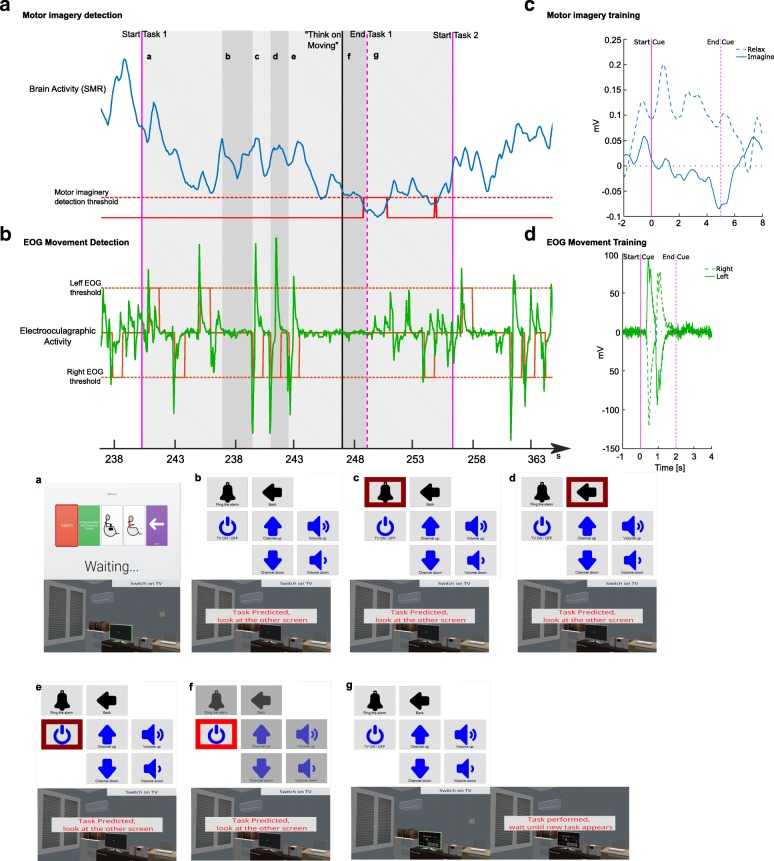
Multimodal system processing for one ADL in *AIDE* mode. The user had to perform different actions in order to execute the corresponding ADLs, in this example, the user had to switch on the TV, phases a-g show the behavior of both screens during the task. EEG (**a**) and EOG (**b**) signals were acquired to online control the ECI in order to perform ADLs in a virtual house. When the task started (vertical purple line), the scan through the ECI was performed by EOG activity detection [orange line in (**b**)], i.e. when HOV activity exceeded the threshold [indicated by the orange dashed line in (**b**)] the grid marker moved forward (phases a-e). Once the subject stopped at one grid, a task confirmation was needed [indicated by the vertical black line] and the ECI ‘switched off’ the rest of the grids indicating this purpose (phase f). The confirmation was performed by the detection of SMR-ERD [indicated by red line in (**a**)] and the action was done, so the ADL finished (vertical dotted purple line). This ADL was performed in one step, i.e. the user only needed to navigate through the last abstraction level to complete the task. Before the experimentation, the user was trained in motor imagery (**c**) and EOG movement (**d**) to the set up the control system with the personalized parameters

Each subject performed two experimental sessions in two consecutive days. The first session was for training and calibration purpose as well as for the familiarization with the systems to be controlled. This session lasted around 60-80 min. In the last part of this session the user learned how to use the hybrid EEG/EOG interface in order to control the ECI. An example of MI and EOG movements in the training session are shown in Fig. 3c and d, respectively.

The second session lasted a maximum of 60 min. The setup and familiarization phase took approximately 15 min (subjects have already tested the system in the first session). They had 10 min to perform a predefined ADLs list in both *AIDE* and *Manual* modes (all ADLs can be observed in Table 1). Each ADL can be a single action, it has a visual effect on the house simulator, e.g. swith on a lamp, or an exoskeleton action, the simulator play a short video showing the corresponding action, e.g. drink from a glass. The order of the modes was randomly selected and, before each mode, a baseline of 3 min was acquired. During the break (5 min) and at the end of this session the subjects answered the NASA task load index (tlx) questionnaire [23].

The scan in the ECI was performed through looking-right eye movements (Fig. 3b) implying a forward displacement of the grid marker (Fig. 3a-e). Once the subject stopped at one grid a customized time, chosen in the first session, the ECI ‘switch off’ the rest of the grids (Fig. 3f). Then, the next level or the action in the ECI was achieved by on-line ERD detection, like the subjects learned during the first session (Fig. 3a). On the other hand, if the user did not want to click on this specific grid, in the phase Fig. 3f, a looking-left eye movement returns the ECI to the phase Fig. 3a. When the user performs an action, a visual feedback is presented in the virtual home regarding to the action performed (Fig. 3g) and he/she waited for the next task.

After both modes, they were instructed to interact with real elements through the ECI and watch a video using objects of a multi-sensory room, as can be observed in Fig. 1b.

## Results

**Fig. 4:**
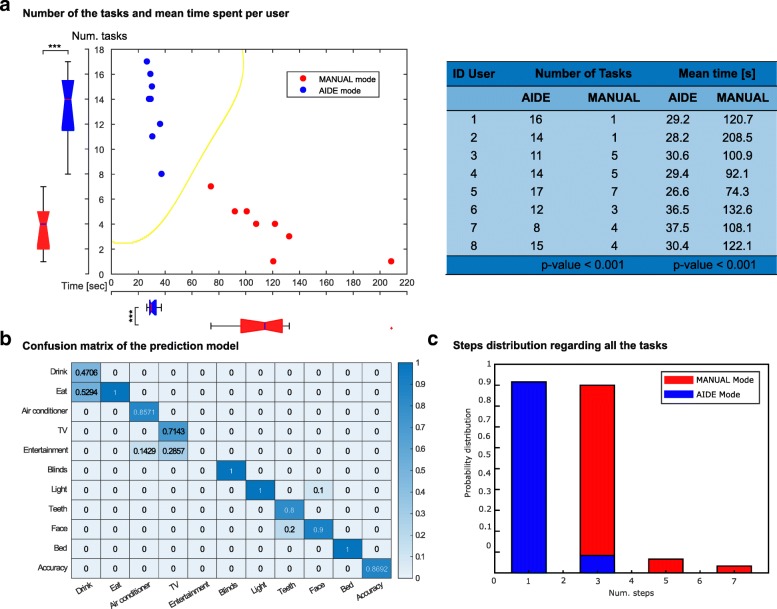
Environment control interface performance. The users performed two different trials with the same goal: complete as many tasks as possible in 10 min by using the *Manual* mode and the *AIDE* mode (**a**). Both modes have been used to train a SVM model with Gaussian kernel and find the boundary between them [yellow line in (**a**)]. The interaction with the ECI was measured by steps that a user had to perform in order to complete the tasks (**c**). Furthermore, in *AIDE* mode the user’s intention prediction was performed through CRF model and, therefore, the confusion matrix of the model is obtained (**b**)

The users performed simulated ADLs during 10 min in a virtual home using an ECI in both *Manual* and *AIDE* modes. The number of the performed tasks with respect to the mean time spent per user is presented in Fig. 4a. Furthermore, it has been trained a Support Vector Machine (SVM) model with Gaussian kernel to estimate the boundary between both modes (yellow line in Fig. 4a). It should be noted that statistically significant differences between both modes in terms of number of tasks and mean tasks time is shown (*p*−*value*<0.001 using Wilcoxon test). The steps distribution that the users performed in both modes are shown in Fig. 4c. ADLs manually omitted tasks were excluded from the study due to the subject was blocked during the ECI scan caused by frustration or fatigue over a specific task.

**Fig. 5:**
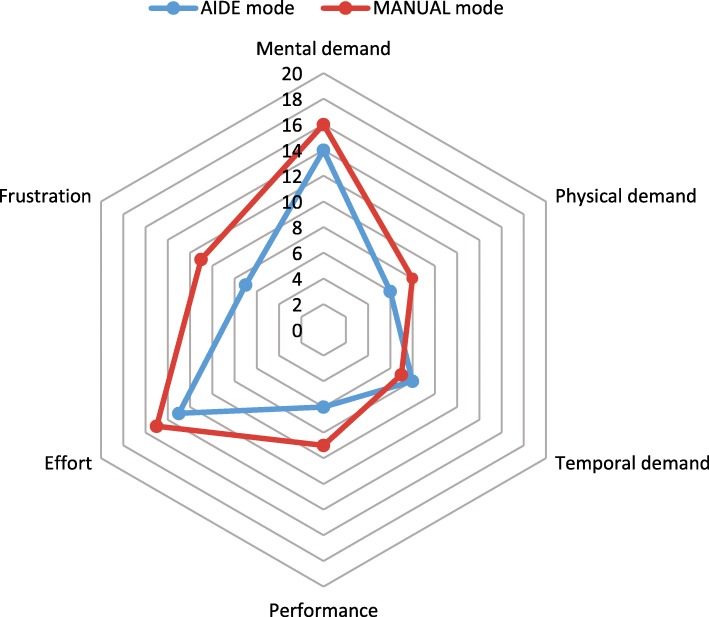
NASA task load index. The subjects answered the NASA tlx questionnaire after each mode

On the other hand, the *AIDE* mode uses a CRF model, previously trained with simulated data using the same virtual home, to predict the user intention. Thus, the confusion matrix of the prediction model regarding the ADL menus is presented in Fig. 4b. In addition, the results obtained through the NASA tlx questionnaire are presented in Fig. 5.

## Discussion

The AIDE project aims to develop a multimodal system in order to help people with neurological diseases wearing a wheelchair. The presented environment allowed the user to navigate through a virtual house and perform several ADLs using a developed ECI. Two modes were estudied, the *AIDE* mode, that used a CRF model to predict the user intention and ease the ECI scan, and the *Manual* mode, that needed a complete scan through the ECI to perform a specific ADL. The ECI was online controlled using the AIDE multimodal system based on a combination of EEG and EOG wireless acquisition system [17].

The results presented in Fig. 4a show the mean time per task spent in the AIDE mode is less than in the Manual mode being able to perform more tasks in the same time, both modes show statistically significant differences (*p*<0.001). Furthermore, both modes can be easily classified by training a SVM model with Gaussian kernel, the boundary is presented with a yellow line in Fig. 4a.

On the other hand, Fig. 4c shows the difference between both groups in terms of steps, i.e. the ECI abstraction levels that the user had to cross in order to perform a specific ADL. It must be noticed that in the *AIDE* mode, the users performed one step in the 90% of the tasks and three steps, at least, were necessary in the *Manual* mode. Regarding the *AIDE* mode, three steps were necessary only when the CRF model realized a wrong prediction and, therefore, the user had to return to the activity menu and select the proper ADL menu. Although a bad prediction is sometimes performed, the multimodal system helps in terms of location, i.e. the activity that the system predicts is always related with the room where the user is, facilitating the navigation. Perform five or seven steps in the *Manual* mode implies that a wrong abstraction level was selected, due to user confusion or lack of practice, and the user had to go back in the ECI.

The *AIDE* mode uses a CRF model to perform the predictions about the user’s intention. The model uses the information of the virtual home and the object that the user is looking at, acquired thought an eye tracking device. The model was previously trained with simulated data using the same virtual house environment. Thus, Fig. 4b shows the confusion matrix of the prediction model, regarding the ADL menu, with a global accuracy about 87%. The CRF model, as it takes into account not only the current state but also the previous states to perform its prediction, it could fail in the prediction of task with common features. Therefore, the ADLs related to the *Drink* menu are sometimes predicted as the ADLs related with the *Eat* menu, in this case around the 50% of the trials. In addition, *TV* and the *Teeth* menus are rarely selected as *Entertainment* and *Face* menu, respectively, by the prediction model.

After each mode, the subjects answered the NASA tlx questionnaire in order to assess the workload between the modes, showed in Fig. 5. Unexpectedly, it has not statistically significant differences, so we can say that the users do not notice subjective differences between both modes in terms of workload. It can be explained because it was the first time that the users handle the complex multimodal control system (EEG+EOG) with this ECI. We assume that, observing the results presented in Fig 4, the workload should decrease, at least, in the AIDE mode.

## Conclusion

The presented ECI allowed the users to perform simulated ADLs with a multimodal control system. The platform was tested in two different scenarios: *Manual* and *AIDE* mode. The first one was presented as a simple ECI where the user had to achieve the corresponding ADL. The second mode used a CRF model to predict the user’s intention through the environment analysis and identification of the user’s behaviors. We conclude that, even the users do not perceive subjective differences between both modes in terms of workload, the *AIDE* mode helps the user to perform mode ADLs, spending less time per task, showing statistically significant differences with respect to the *Manual* mode. This effect is caused by the user’s intention prediction as the ECI jumps directly to the last abstraction level of the ECI. The environment analysis and the identification of the user’s behaviors can be used to predict the user intention and will allow to speed up the ECIs scan opening a new paradigm in the design of these interfaces. Although the developed ECI was tested only in a simulated home environment, it can be easily adapted to a real environment increasing the user independence at home.

## Abbreviations

AAC: Augmentative and alternative communication
ADL: Activity of daily living
BCI: Brain computer interface
CRF: Conditional random field
ECI: Environment control interface
EEG: Electroencephalography
EOG: Electrooculography
HOV: Horizontal oculoversion
MI: Motor Imagery
SMR-ERD: Sensorimotor rhythm event-related desynchronization
SVM: Support vector machine
tlx: task load index

## Footnotes

1
http://aideproject.umh.es/

2
http://www.thinksmartbox.com

3
http://www.tobii.com

## References

[1] World Health Organization. Neurological Disorders: Public Health Challenges.Geneva: World Health Organization; 2006. http://www.who.int/iris/handle/10665/43605.

[2] Winstein CJ, Stein J, Arena R, Bates B, Cherney LR, Cramer SC, Deruyter F, Eng JJ, Fisher B, Harvey RL (2016). Guidelines for adult stroke rehabilitation and recovery: a guideline for healthcare professionals from the american heart association/american stroke association. Stroke.

[3] Sellers EW, Vaughan TM, Wolpaw JR (2010). A brain-computer interface for long-term independent home use. Amyotroph Lateral Scler.

[4] Ball MM, Perkins MM, Whittington FJ, Hollingsworth C, King SV, Combs BL (2004). Independence in assisted living. J Aging Stud.

[5] Ball MM, Perkins MM, Whittington FJ, Connell BR, Hollingsworth C, King SV, Elrod CL, Combs BL (2004). Managing decline in assisted living: The key to aging in place. J Gerontol B Psychol Sci Soc Sci.

[6] Struijk LNA, Egsgaard LL, Lontis R, Gaihede M, Bentsen B (2017). Wireless intraoral tongue control of an assistive robotic arm for individuals with tetraplegia. J Neuroengineering Rehabil.

[7] Brandt Å, Samuelsson K, Töytäri O, Salminen A-L (2011). Activity and participation, quality of life and user satisfaction outcomes of environmental control systems and smart home technology: a systematic review. Disabil Rehabil Assist Technol.

[8] Agree EM, Freedman VA, Cornman JC, Wolf DA, Marcotte JE (2005). Reconsidering substitution in long-term care: when does assistive technology take the place of personal care?. J Gerontol B Psychol Sci Soc Sci.

[9] O’Neill B, Moran K, Gillespie A (2010). Scaffolding rehabilitation behaviour using a voice-mediated assistive technology for cognition. Neuropsychol Rehabil.

[10] Lupu RG, Ungureanu F, Siriteanu V. Eye tracking mouse for human computer interaction. In: E-Health and Bioengineering Conference (EHB), 2013. IEEE: 2013. p. 1–4. 10.1109/EHB.2013.6707244.

[11] Adjouadi M, Sesin A, Ayala M, Cabrerizo M (2004). Remote eye gaze tracking system as a computer interface for persons with severe motor disability. International Conference on Computers for Handicapped Persons.

[12] Biswas P, Langdon P (2011). A new input system for disabled users involving eye gaze tracker and scanning interface. J Assist Technol.

[13] Miralles F, Vargiu E, Rafael-Palou X, Sola M, Dauwalder S, Guger C, Hintermuller C, Espinosa A, Lowish H, Martin S, Armstrong E, Daly J (2015). Brain?computer interfaces on track to home: Results of the evaluation at disabled end-users? homes and lessons learnt. Front ICT.

[14] Wolpaw JR, Birbaumer N, McFarland DJ, Pfurtscheller G, Vaughan TM (2002). Brain–computer interfaces for communication and control. Clin Neurophysiol.

[15] Cincotti F, Mattia D, Aloise F, Bufalari S, Schalk G, Oriolo G, Cherubini A, Marciani MG, Babiloni F (2008). Non-invasive brain–computer interface system: towards its application as assistive technology. Brain Res Bull.

[16] Witkowski M, Gómez C, Opisso E, Medina J, Cortese M, Cempini M, Carrozza MC, Cohen LG, Birbaumer N, Vitiello N (2016). Hybrid eeg/eog-based brain/neural hand exoskeleton restores fully independent daily living activities after quadriplegia. Sci Robot.

[17] Soekadar SR, Witkowski M, Mellinger J, Ramos A, Birbaumer N, Cohen LG (2011). Erd-based online brain-machine interfaces (bmi) in the context of neurorehabilitation: optimizing bmi learning and performance.. IEEE Trans Neural Syst Rehabil Eng: Publ IEEE Eng Med Biol Soc.

[18] Lafferty J, McCallum A, Pereira FC (2001). Conditional random fields: Probabilistic models for segmenting and labeling sequence data. Proceedings of the Eighteenth International Conference on Machine Learning (ICML ’01).

[19] Schalk G, McFarland DJ, Hinterberger T, Birbaumer N, Wolpaw JR (2004). Bci2000: a general-purpose brain-computer interface (bci) system. IEEE Trans Biomed Eng.

[20] Barios JA, Ezquerro S, Bertomeu-Motos A, Nann M, Badesa FJ, Fernandez E, Soekada SR, Garcia-Aracil N. Synchronization of slow cortical rhythms during motor imagery-based brain-machine interface control. Int J Neural Syst. 2018. in press. 10.1142/S0129065718500454 30587046

[21] Rabiner LR (1989). A tutorial on hidden Markov models and selected applications in speech recognition. Proc IEEE.

[22] Quinn TJ, Langhorne P, Stott DJ (2011). Barthel index for stroke trials: development, properties, and application. Stroke.

[23] Hart SG, Staveland LE (1988). Development of nasa-tlx (task load index): Results of empirical and theoretical research. Advances in Psychology, vol 52.

